# A qPCR-based metric of Th2 airway inflammation in asthma

**DOI:** 10.1186/2045-7022-3-24

**Published:** 2013-07-17

**Authors:** Nirav R Bhakta, Owen D Solberg, Christine P Nguyen, Cindy N Nguyen, Joseph R Arron, John V Fahy, Prescott G Woodruff

**Affiliations:** 1 Cardiovascular Research Institute, University of California, San Francisco, USA; 2 Department of Medicine, Division of Pulmonary, Critical Care, Sleep and Allergy, University of California, Room HSE-1355A, 513 Parnassus Avenue, Box 0130, San Francisco, CA 94143, USA; 3 Genentech, Inc, South San Francisco, CA, USA

**Keywords:** Asthma, Inflammation, Endophenotypes (all MeSH terms), Biomarkers, Th2

## Abstract

**Background:**

Using microarray profiling of airway epithelial cells, we previously identified a Th2-high molecular phenotype of asthma based on expression of periostin, CLCA1 and serpinB2 and characterized by specific inflammatory, remodeling, and treatment response features. The goal of the current study was to develop a qPCR-based assay of Th2 inflammation to overcome the limitations of microarray-based methods.

**Methods:**

Airway epithelial brushings were obtained by bronchoscopy from two clinical studies comprising 44 healthy controls and 62 subjects with asthma, 39 of whom were studied before and after a standardized 8 week course of inhaled corticosteroids (ICS). The qPCR-based expression of periostin, CLCA1 and serpinB2 were combined into a single metric.

**Results:**

In asthma, the three-gene-mean of periostin, CLCA1 and serpinB2 correlated with FeNO (r = 0.75, p = 0.0002), blood eosinophils (r = 0.58, p = 0.003) and PC_20_ methacholine (r = -0.65, p = 0.0006), but not total serum IgE (r = 0.33, p = 0.1). Higher baseline three-gene-mean correlated with greater improvement in FEV_1_ with ICS at 2, 4 and 8 weeks (all p < 0.05). By ROC analysis, the area under the curve (AUC) of the three-gene-mean for FEV_1_ improvement with ICS at 4 and 8 weeks was 0.94 and 0.87, respectively, which are higher than the AUCs of FeNO, blood eosinophils, IgE or PC_20_. Th2 airway inflammation as measured by this three-gene-mean also had predictive capacity for an improvement in symptoms.

**Conclusions:**

The three-gene-mean of periostin, CLCA1 and serpinB2 in airway epithelial brushings identifies Th2-high and low populations, is correlated with other Th2 biomarkers, and performs well for prediction of FEV_1_ improvement with ICS. The three-gene-mean provides a measurement of Th2 airway inflammation that is clinically relevant and that can serve as a valuable tool to evaluate non-invasive biomarkers to predict treatment responses to existing and emerging asthma therapies.

## Background

Asthma is heterogeneous, and more refined phenotypic subgroupings will be required to better guide clinical management [1]. For example, serum IgE levels are used to successfully target the anti-IgE monoclonal antibody omalizumab [2], and sputum eosinophils have been used to target the anti-IL5 antibody mepolizumab [3-5]. Nonetheless, most asthma guidelines do not account for underlying biological mechanisms in the decision to treat with the most commonly used anti-inflammatory therapy, inhaled corticosteroids (ICS). Indeed, randomized controlled trials have shown that 30-45% of patients with asthma fail to have an improvement in FEV_1_ even to high dose ICS [6,7], and 25% are not well-controlled based upon symptoms [8]. These data suggest that biomarkers are needed to determine who will benefit from ICS or from progressive increases in ICS dosing. Furthermore, as targeted therapies such as anti-IgE and anti-interleukin-13 become more widely available, the identification of the patients who are most likely to respond will be critical.

Our group has previously identified airway epithelial gene expression markers of a Th2-high molecular phenotype of asthma [9]. In a clinical trial of ICS, only this Th2-high subgroup had the expected increase in FEV_1_. However, one practical limitation is that Th2 status was established through unsupervised hierarchical clustering of microarray data [9]. Disadvantages inherent to clustering of microarray data include instability in phenotype assignment, cost, inefficient use of RNA, and only a dichotomous high vs low output. These limitations have been partially addressed by the development of a quantitative gene expression signature from airway biopsies, but this metric still was constrained by the use of microarray data [10]. Another limitation of our prior work is that we did not concurrently measure the fraction of exhaled nitric oxide (FeNO), an alternative biomarker of Th2 inflammation. Our overarching goal in the current study was to develop a continuous metric of Th2 inflammation based on qPCR that avoids the limitations of array- and cluster-based phenotyping and that can be used to standardize the measurement of Th2 inflammation across bronchoscopy-based asthma studies. Our specific goals were to determine the relationship between our qPCR-based Th2 metric and other accepted markers of Th2 inflammation including FeNO, and ascertain how well this metric predicts lung function and symptom response to ICS when compared to other candidate biomarkers.

## Methods

### Research participants

We studied two cohorts that were recruited sequentially at UCSF. Our primary analyses were performed on the more recent cohort (hereafter referred to as the primary cohort) which has not previously been studied by mRNA microarray or qPCR for Th2 phenotyping (miRNA data are published elsewhere [11]). Additional analyses were performed in a secondary cohort in which we have previously performed microarray-based Th2-phenotyping [9]. See Additional file 1: Figure S1 for additional details on these cohorts. The primary cohort comprised adults aged 18-70 years recruited via community-based advertising into two groups: 1) 22 healthy controls and 2) 32 subjects with mild-to-moderate asthma not on ICS. Healthy controls had no history of asthma or allergic rhinitis. Subjects with asthma had a participant-reported history of asthma. Subjects with asthma were also required to not have used inhaled or oral steroids for at least 6 weeks prior to enrollment, to have airway hyper-responsiveness (AHR) to methacholine (PC_20_ to methacholine < = 8.0 mg/mL), and at least one of the following: asthma symptoms on at least two days per week, beta agonist use on at least two days per week, or FEV_1_ < 85% of predicted. All participants were non-smokers defined as never smoker or former smokers with no smoking for at least 1 year *and* total pack-years < = 15. Additional exclusion criteria were pregnancy, upper respiratory tract infection in the 4 weeks prior to enrollment, and the use of certain other medications [11].

### Brief description of the secondary cohort

In this report, the secondary cohort contributes 25 healthy controls and 38 subjects with mild asthma pre-ICS, of which 18 received 8 weeks of ICS. The research protocol is similar to that for the primary cohort [9], with the following relevant differences: 1) FeNO and symptoms were not measured; 2) ICS treatment was with fluticasone 500 μg inhaled twice daily; 3) a second bronchoscopy was performed at the end of 1 week after the initiation of ICS, rather than at the end of 8 weeks as in the primary cohort.

### Research protocol in the primary cohort

Baseline characterization included detailed medical history and examination, spirometry, PC_20_ to methacholine (highest concentration used: 10 mg/ml), measurement of FeNO (NIOX MINO, Aerocrine), complete blood count and differential and measurement of serum total IgE. On the second study visit (1-2 weeks following the baseline visit) subjects underwent bronchoscopy and subjects with asthma were then treated with inhaled budesonide 180 μg twice daily for 8 weeks; subjects maintained diaries of symptoms (shortness of breath, chest tightness, wheeze, cough, or sputum) and returned for spirometry at 2, 4 and 8 weeks. At 8 weeks, subjects underwent repeat bronchoscopy and blood draw for IgE and eosinophil measurements. All clinical studies were approved by the University of California at San Francisco Committee on Human Research, written informed consent was obtained from all subjects, and all studies were performed in accordance with the principles expressed in the Declaration of Helsinki. The study was registered at Clinicaltrials.gov (NCT00595153).

### PCR

RNA extraction and two-step, nested-primer qPCR was performed as previously described [12]. Epithelial brushings obtained during research bronchoscopy were stored in RLT, with subsequent RNA extraction using column-based purification (Rneasy Micro, Qiagen). RNA quantity was determined by a Nanodrop spectrophotometer (Thermo). RNA quality was assessed on an Agilent Bioanalyzer. 20 ng of RNA was reverse transcribed with random hexamer primers (Superscript/VILO, Invitrogen), and then amplified in multiplex for POSTN, CLCA1, SERPINB2, and endogenous control genes (EEF1A1, PPIA, RPL13A, DNAJA1, and ACTB in the primary cohort; EEF1A1, PPIA, RPL13A in the secondary cohort) with custom primers (Biosearch Technologies; Additional file 1: Table S1) and the Advantage polymerase and reaction mix (Clontech). The resulting amplified cDNA underwent uniplex qPCR with custom primers and Taqman-based probes (Biosearch Technologies; Additional file 1: Table S1), with samples run across separate batches (on a 7900 or Viia 7 from ABI). Potential batch effects were corrected by adjusting cycle threshold (Ct) values on a per-gene basis using samples that were replicated across all three batches, using methods described previously [13]. For each gene, log_2_-transformed, normalized, relative expression values were generated by subtracting the Ct value for a particular sample from the mean Ct of the control genes (only EEF1A1, PPIA, RPL13A were used for comparability of qPCR data between the primary and secondary cohorts) for that sample. This approach assumes equal PCR efficiency for all genes of 100%, and produces a result that is mathematically equivalent to normalization of expression values by the geometric mean expression of the control genes [14]. Relative, normalized, transcript quantities were log_2_-transformed and then centered and scaled. The Th2 metric is the mean of the centered and scaled values for periostin, serpinB2, and CLCA1 and is labeled the “three-gene-mean”.

### Statistical methods

Statistical analyses were performed in R version 2.15.1 [15]. Correlations used Pearson's sample correlation coefficient. Univariate tests used a Welch's *t* test (for unequal variances) unless otherwise noted. Total serum IgE, blood eosinophil concentrations, FeNO, and PC_20_ to methacholine were log-transformed prior to analysis. ROC curves were computed using the *pROC* package [16]. All plots were created with the *ggplot2* package [17]. p < 0.05 was taken as statistically significant, without corrections for multiple testing.

## Results

### Study subjects in the primary cohort

Of 22 eligible healthy controls and 32 eligible subjects with asthma, 20 and 26, respectively, underwent bronchoscopy; 23 subjects with asthma underwent repeat bronchoscopy at the end of 8 weeks. Data in this report are from the 19 healthy controls, 24 subjects with asthma pre-ICS, and 21 subjects post-ICS from whom epithelial brushings and qPCR data were obtained (Table 1). FEV_1_ and the ratio of FEV_1_ to FVC were lower in subjects with asthma compared to healthy controls but both groups had FEV_1_ > 80% predicted (Table 1). Subjects with asthma had increases in multiple markers of inflammation and atopy: FeNO, total serum IgE, and blood eosinophils.

**Table 1:** Baseline subject characteristics

	**Healthy controls**	**Asthmatics**	**p value**
Sample size of qPCR data	19	24	
Age, years	34 ± 9	33 ± 12	0.71
BMI	26 ± 4	28 ± 5	0.20
Gender (% F)	42	50	0.84
Ethnicity*			
White	10	10	
African-American	1	2	
Hispanic	3	6	
Asian/Pacific Islander	4	5	
Native American	1	1	
FEV_1_, % predicted	99 ± 13	84 ± 13	0.0008
Δ FEV_1_ with albuterol (% of baseline)	5.0 ± .4.5	14.4 ± 9.5	0.0001
FEV_1_/FVC	0.78 ± 0.066	0.73 ± 0.074	0.02
Methacholine PC_20_ (mg/ml)	> 10 (n = 18)	0.57 (0.07-4.40)	<0.0001
IgE, IU/ml	20 (4–357), (n = 18)	232 (2–6556)	<0.0001
Blood eosinophils, ×10^9^/L	0.11 (0.04-0.30)	0.33 (0.05-0.75)	<0.0001
FeNO	16 (9–49)	62 (9–144), (n = 18)	0.0004

Values reported as mean ± standard deviation, or median (range). All data are from the baseline visit except spirometry, which are reported from visit 2 (immediately before initiation of ICS). p- values are based on *t*-test or chi-square test for proportions unless otherwise noted.

* In cases of multiple ethnicities, the lowest-numbered ethnicity from this list is reported: 1. Hispanic, 2. Black, 3. Pacific Islander, 4. Native American, 5. Asian, 6. White.

### qPCR assay to determine the degree of Th2 inflammation

**Figure 1:**
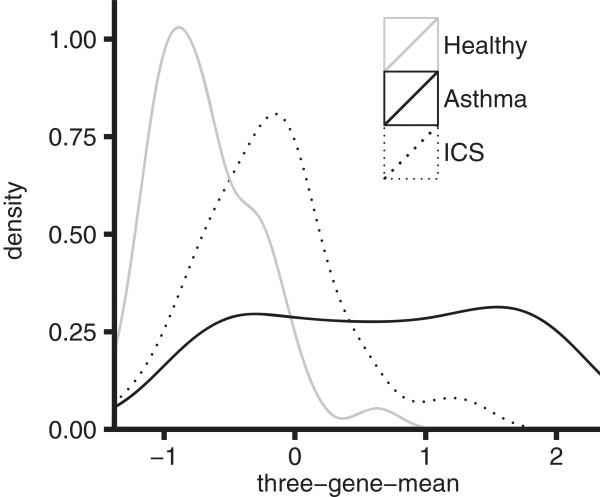
**Distribution of airway epithelial metric of Th2 inflammation: three-gene-mean values.** Smoothed distributions of three-gene-mean values across both the primary study (n = 19 healthy controls, 24 subjects with asthma not on ICS, and 21 subjects with asthma subsequently treated with ICS—budesonide 180 μg twice daily for 8 weeks) and secondary study (n = 25 healthy controls, 38 subjects with asthma not on ICS, and 18 subjects with asthma subsequently treated with ICS—fluticasone 500 μg twice daily for 8 weeks).

In our prior study, Th2-high vs low phenotype was assigned through hierarchical clustering which separated groups on the basis of the microarray expression of three epithelial-derived markers of Th2-driven inflammation: CLCA1, periostin, and serpinB2 [9]. In the current study, we developed a PCR-based assay for the same three genes in epithelial brushings. The expression levels of these three genes were intercorrelated across healthy subjects and subjects with asthma (Additional file 1: Figure S2). Given our aim to create an accurate measure of the degree of Th2-driven inflammation present in the airway, this intercorrelation suggested that the effect of variation in individual genes (e.g. changes not in response to Th2 cytokines) would be mitigated by combining their expression values into a single metric. Thus, the expression values for these three genes were averaged into a single quantitative metric we call the “three-gene-mean”, as described in the Methods. The distribution of the three-gene-mean for subjects with asthma not on ICS across both primary and secondary cohorts has two peaks suggesting a natural separation between subjects with low and high levels of Th2 inflammation (Figure 1); nonetheless, it is clear there are a considerable number of subjects with asthma with intermediate levels of Th2 inflammation as well. The peak corresponding to lower values is slightly shifted to the right of the healthy distribution, indicating that as a group, even asthmatic airways with low Th2 inflammation have more Th2 activity than healthy airways. In subjects with asthma on ICS, there is an incomplete reduction of Th2 airway inflammation towards healthy values as measured by this metric (Figure 1).

### Relationship of three-gene-mean to FeNO and other Th2-markers

**Figure 2:**
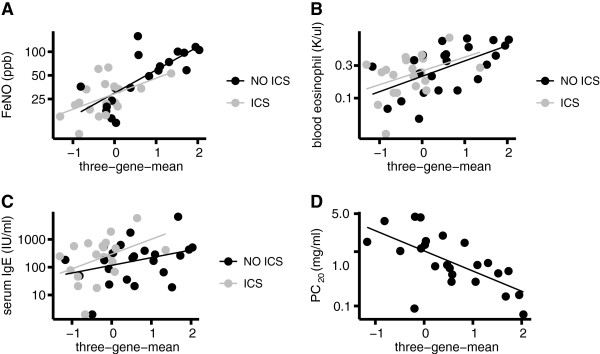
**Relationship of baseline three-gene-mean to other markers of Th2 airway inflammation and ICS response.** Three-gene-mean values were measured from airway epithelial brushings acquired at bronchoscopy before and after 8 weeks of ICS. Covariates were also measured before and after ICS, and log-transformed to yield normally distributed values prior to statistical calculations. Scatterplots of covariates (on a log_10_ scale) vs three-gene-mean are shown with least-squares linear regression lines before (black) and after (gray) ICS for the following variables: **A**. Exhaled nitric oxide (FeNO), r = 0.75, p = 0.00023 off ICS; r = 0.53, p = 0.024 on ICS. **B**. Blood eosinophils, r = 0.58, p = 0.003; r = 0.40, p = 0.071 on ICS. **C**. Total serum IgE, r = 0.33, p = 0.11 off ICS; r = 0.42, p = 0.067 on ICS. **D**. PC_20_ to methacholine, r = -0.65, p = 0.0006 off ICS; PC_20_ not obtained after ICS treatment.

The three-gene-mean correlates extremely well with FeNO both in the absence of ICS (r = 0.75, p = 0.0002), and in the presence of ICS (r = 0.53, p = 0.024) (Figure 2A). The three-gene-mean is also significantly correlated with blood eosinophils in subjects with asthma not using ICS (r = 0.58, p = 0.003) but this relationship is not significant in subjects on ICS (r = 0.40, p = 0.07) (Figure 2B). Whether on or off ICS, the relationship between Th2 airway inflammation and total serum IgE was not significant (r = 0.33, p = 0.1 off ICS; r = 0.42, p = 0.07 on ICS; Figure 2C).

### Relationship of three-gene-mean to airway hyper-responsiveness

We found a highly significant correlation between the three-gene-mean and PC_20_ methacholine measured at baseline off ICS, with 43% of the variance in PC_20_ explained by the degree of Th2 inflammation (r = -0.65, p = 0.0006; Figure 2D). This suggests that in people with asthma, the degree of Th2 inflammation is a significant determinant of AHR, the functional abnormality of airway smooth muscle which is characteristic of asthma.

### Relationship of three-gene-mean to lung function improvement with ICS

**Figure 3:**
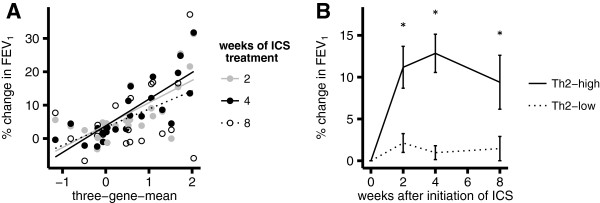
**Relationship between FEV**_**1 **_**changes in response to ICS and baseline Th2 airway inflammation in asthma.** Percent change in pre-bronchodilator FEV_1_ is relative to week 0 (baseline). **A**. Percent change in FEV_1_ at 2 (gray solid circles), 4 (black solid circles) and 8 (open circles) weeks, versus Th2 airway inflammation measured by the three-gene-mean at baseline, and associated least-squares linear regression lines. **B**. Mean percent change in FEV_1_ stratified by a dichotomous Th2 grouping, with Th2-high defined as a three-gene-mean greater than a value of 0.1, as suggested by the midline between the peaks for subjects with asthma not on ICS in Figure 1. Dotted and solid lines represent Th2-low and Th2-high subjects respectively. Error bars are standard error of the mean. * statistically significant (see text).

Higher baseline three-gene-mean was associated with greater improvement in lung function with ICS treatment at 2 weeks (r = 0.68, p = 0.0004), 4 weeks (r = 0.79, p = 7×10-6) and 8 weeks (r = 0.47, p = 0.03) (Figure 3A). In further analyses, we dichotomized Th2 status at 2 standard deviations above the mean of the healthy distribution for three-gene-mean (Th2-high defined as > 0.1). The mean percent change in FEV_1_ from baseline was significantly higher in Th2-high compared to Th2-low asthma at 2 weeks (difference of 9%, p = 0.004), 4 weeks (difference of 12%, p = 0.0002) and 8 weeks (difference of 8%, p = 0.038) (Figure 3B; individual subject responses in Additional file 1: Figure S3). As an alternative approach, we dichotomized based upon the natural separation in the three-gene-mean suggested by Figure 1 (Th2-high defined as baseline three-gene-mean > 0.5). With this alternate threshold, the mean change in FEV_1_ was significantly higher in Th2-high asthma subjects at 2 weeks (difference of 10%, p = 0.006) and 4 weeks (difference of 13%, p = 0.0001), with a similar trend at 8 weeks (difference of 8%, p = 0.055).

**Figure 4:**
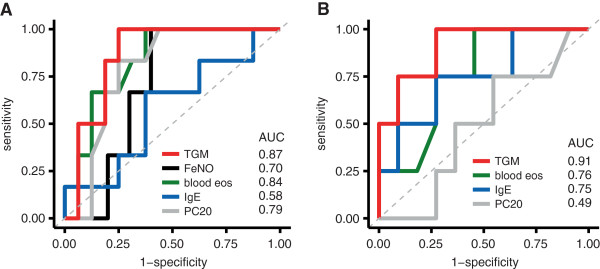
**Predictive performance of three-gene-mean and other markers of Th2 inflammation for 8 week ICS response.** Receiver operating characteristic (ROC) curves were made to assess the ability of the three-gene-mean and other markers to predict an improvement in FEV_1_ after 8 weeks of ICS. Improvement in FEV_1_ was defined as an increase of at least 200 ml and 12% relative to week 0. **A**. ROC curves for baseline measurements of the three-gene-mean (labeled “TGM”), FeNO, blood eosinophils, total serum IgE, and PC_20_ to methacholine in the primary cohort. Six out of 22 subjects had an improvement in FEV_1_. **B**. ROC curves for the secondary cohort to validate the findings in panel **A**. Four out of 15 subjects had an improvement in FEV_1_. In both panels, the line of unity is represented by a dashed gray line, corresponding to an AUC of 0.5.

To evaluate the sensitivity and specificity of the three-gene-mean for prediction of ICS response, we performed an ROC analysis. We defined a positive FEV_1_ response as an increase of ≥12% and ≥200 ml, paralleling ATS recommendations [18]. Six out of 22 asthma subjects had a positive response to ICS at 8 weeks (7 out of 23 at 4 weeks). The area under the curve (AUC) of baseline three-gene-mean for prediction of FEV_1_ response was 0.87 at 8 weeks (Figure 4A) and 0.94 at 4 weeks (Additional file 1: Figure S4A). Validation in the secondary cohort (4 responders out of 15 subjects at 8 weeks) confirmed a high AUC for three-gene-mean with a value of 0.91 at 8 weeks (Figure 4B) and 0.77 at 4 weeks (Additional file 1: Figure S4B). Table 2 summarizes the quantitative performance of the three-gene-mean by ROC analysis.

**Table 2:** Summary of ROC analysis for three-gene-mean

	**Primary cohort**		**Secondary cohort**	
	**4 weeks**	**8 weeks**	**4 weeks**	**8 weeks**
AUC	0.94	0.87	0.77	0.91
Sensitivity (%) three-gene-mean threshold*				
0.1	100	100	100	100
0.5	100	100	100	100
0.57	100	100	75	100
Specificity (%) three-gene-mean threshold*				
0.1	56	50	33	27
0.5	69	63	42	36
0.57	81	75	42	45

* For the baseline three-gene-mean thresholds, the cutoff of 0.5 is suggested by the two peaks in Figure 1, the cutoff of 0.1 is derived from the mean + 2SD of the healthy distribution, and the cutoff of 0.57 maximizes the sum of sensitivity and specificity of the three-gene-mean in the primary cohort at 8 weeks.

Given our aim to create a gold-standard of Th2 airway inflammation with the three-gene-mean, we next compared this metric's performance to that of classical markers of allergic inflammation and AHR, which as shown above and summarized in Additional file 1: Table S2, were well correlated with the three-gene-mean with the exception of total serum IgE. Although our datasets are underpowered to perform a multivariate comparison as with logistic regression, we found it informative to compare performance with ROC analysis. The AUC for three-gene-mean at 8 weeks was greater than the AUC for FeNO, blood eosinophils and IgE (Figure 4A). In addition, although AHR is not considered a Th2-specific characteristic per se, measurement of AHR by methacholine challenge has been shown to be a valuable guide in adjustment of ICS dosing as compared to a standard guideline-based algorithm in a prior study [19]. We found that the AUC for prediction of ICS-response was better for the three-gene-mean than for PC_20_ methacholine (Figure 4A). Validation in the secondary cohort (4 responders out of 15 subjects at 8 weeks) showed that the three-gene-mean again had a higher AUC compared to other potential biomarkers of ICS response (Figure 4B at 8 weeks; Additional file 1: Figure S4B at 4 weeks).

Finally, whereas an individual gene component may have a higher AUC than the three-gene-mean in some instances, the three-gene-mean has more consistent performance across the datasets (Additional file 1: Table S3). This consistency in the three-gene-mean led us to examine whether the mean of the three markers with which it was best correlated with—blood eosinophils, FeNO, and PC_20_—may also perform well in predicting ICS response. The results in Additional file 1: Table S3 show AUC's for this combined non-invasive metric that are comparable to, albeit lower than those for the three-gene-mean.

### Th2 inflammation and symptom improvement with ICS

Using a baseline three-gene-mean cutoff value of 0.1, subjects with Th2-low asthma did not have significantly different baseline percentage of days with symptoms (defined as any shortness of breath, chest tightness, wheeze, cough, or sputum) compared to those with Th2-high asthma (82% for Th2-high, 78% for Th2-low, p = 0.7). However, subjects with Th2-high asthma did have a significantly greater improvement in the percentage of days with symptoms compared to Th2-low asthma subjects after 4 weeks of ICS (mean absolute decrease of 42% vs 10%, p = 0.03), and a trend at 8 weeks (mean absolute decrease of 53% vs 24%, p = 0.08). Without an accepted threshold to dichotomize the response in symptoms, we did not perform ROC analyses to compare predictors.

## Discussion

This qPCR-based three-gene-mean metric contributes to our understanding of Th2 inflammation in asthma by: 1) demonstrating that previously published epithelial gene expression markers correlate well with FeNO, 2) identifying a strong association between Th2 inflammation and smooth muscle dysfunction (measured by AHR) among patients with asthma, and 3) demonstrating that epithelial gene expression markers have good predictive capacity for both lung function and symptomatic improvement with ICS. Since it is obtained through bronchoscopic evaluation, the major application of this three-gene-mean metric of Th2 inflammation will be in clinical and translational research studies. However, development of a quantitative and reproducible gold-standard for clinical research applications is a necessary pre-requisite for the assessment of non-invasive biomarkers.

This qPCR-based metric has several advantages over our prior classification which used hierarchical clustering of microarray data. Most notably, microarrays and hierarchical clustering cannot be repeated in a standardized way in future studies. This new metric of Th2 airway inflammation requires as little as 20 ng of RNA from airway epithelial cells using qPCR, a widely available technique. With the use of inter-run calibrators, three-gene-mean values can be compared across different studies, platforms, reagents, and laboratories [20]. Finally, a continuous measurement of Th2 airway inflammation provides more statistical power to detect associations with other clinical and molecular features of asthma, without losing the ability to apply a threshold to make dichotomous Th2-high vs low classifications for practical applications. An important finding from application of this continuous metric is that regardless of where the threshold is made, Th2-low asthma subjects have low rather than absent Th2 inflammation. Analysis of microarray-based gene expression in airway biopsies from our earlier study also showed that a continuum of Th2 inflammation is present even among those with Th2-low inflammation [10].

To place our metric into context, we correlated the three-gene-mean with a range of other markers of Th2 inflammation and ICS responsiveness. We found a strong correlation between the three-gene-mean and blood eosinophils, FeNO, and PC_20_ methacholine, but not with total serum IgE. In particular, FeNO was well correlated with the three-gene-mean both before and after a course of ICS, suggesting that FeNO measurements are a good surrogate for the three-gene-mean. Nitric oxide production is increased in the lungs of asthma patients in part due to increased transcription of the NOS2 gene encoding inducible nitric oxide synthase in epithelial cells [21]. We have previously found airway epithelial expression of periostin, CLCA1, and serpinB2 to be correlated with NOS2 expression [10,22]. However, we have not previously directly compared our epithelial expression markers to FeNO. FeNO's reported ability to identify a subgroup of severe asthmatics on high-dose ICS that selectively respond to the Th2-targeted, anti-interleukin-13 monoclonal antibody lebrikizumab, also supports its strength as a Th2 marker [22,23]. Notably, however, FeNO has not performed well in clinical studies as a measure by which to modulate ICS dose, which may be from poor reproducibility and other technical issues [22,24]. Therefore, the identification of additional non-invasive markers of Th2 inflammation may be valuable. Sputum eosinophils have also been proposed as a marker of Th2 airway inflammation, with studies showing that modulating ICS therapy by sputum eosinophils counts can lead to a reduced number and severity of exacerbations [24,25]. Unfortunately, we did not have sputum eosinophil counts in the present study, but certainly its relationship to the three-gene-mean will be of interest in future studies.

Importantly, sputum eosinophil measurements are difficult to perform in clinical practice; an easier alternative is protein levels of serum periostin, which like FeNO, identified a subgroup of severe asthmatics responsive to lebrikizumab [22,23]. Although serum periostin was not measured in the current study, future studies that relate the three-gene-mean to serum periostin will potentially help optimize the threshold for serum periostin that best identifies individuals that will respond to ICS or Th2-targeted therapies. Such an analysis will also provide a better understanding of the relationship between serum periostin and Th2 airway inflammation, especially in the setting of other, non-asthma epithelial disorders that can potentially alter serum periostin levels such as allergic diseases of the gastrointestinal tract, skin, and upper airway.

Using ROC curves, we compared the performance of the three-gene-mean to other markers of Th2 inflammation in predicting response to treatment with ICS. The more consistent performance for the three-gene-mean across our two datasets compared to its components supports the concept that the mean may be resistant to individual gene-level variation that is less reflective of Th2-driven inflammation. The three-gene-mean had a larger AUC than FeNO, eosinophils, total serum IgE, PC_20_, and a combination of three non-invasive measures (FeNO, eosinophils, and PC_20_) for FEV_1_ improvement. Overall, this comparative performance analysis suggests that non-invasive measures can accurately predict ICS response, and that the three-gene-mean is a valid research tool by which to establish a lung-based gold-standard for evaluating other biomarkers of Th2 inflammation.

Airway epithelial cells are increasingly recognized as a source of key initiators of allergic inflammation including IL-33, IL-25, and TSLP [26]. Therefore, it is possible that transcript levels of these genes may also serve as useful biomarkers of allergic inflammation. Although we did not measure the expression levels of these genes by qPCR in this study to test this particular hypothesis, genome-wide microarray expression profiling on the cohorts in this study shows no difference between subjects with asthma and healthy controls for IL-33, IL-25, and TSLP (data not shown). Whether these negative results are due to limitations of the probes on the microarray or true lack of transcript-level differences can be addressed in a future study.

As an alternative to biomarkers, published reports have identified lung function measures as predictors of steroid response in asthma. Baseline values for FEV_1_ as percent of predicted, the FEV_1_/FVC ratio, and bronchodilator percent reversibility were strongly correlated with improvement in FEV_1_ in response to ICS [6,7]. All three of these spirometry-based predictors use baseline FEV_1_, which is a parameter used in the calculation of the outcome, improvement in FEV_1_. Therefore, the predictive value of these lung function measures for lung function improvement is in part tautological. In addition, biological markers have the advantage over lung function measures that these markers can identify the presence of specific inflammatory pathways targeted by specific therapeutics. The most salient examples would be Th2-targeted biologics such as mepolizumab and lebrikizumab.

Th2 sub-grouping of asthma has broad clinical research applications. The presence of features of asthma despite low levels of Th2 inflammation raises the question of whether other underlying abnormalities are present in the airways of patients with Th2-low asthma. Hypotheses proposed for the pathology in Th2-low asthma include intrinsic airway smooth muscle dysfunction and non-Th2 inflammatory pathways such as Th17-driven inflammation [27,28]. The ability to characterize a patient with asthma as Th2-low and study airway specimens from this subgroup enables testing of these proposed underlying abnormalities. It is likely that some individuals with Th2-high asthma also have non-Th2 pathways of inflammation active given that Th2 status only partially explains asthmatic features such as airway mucin stores and AHR [9]. Assignment of Th2 status to research subjects will also allow the study of abnormalities that co-exist with Th2 inflammation.

We recognize a number of limitations in the current study. The sample size did not prevent validation of the three-gene-mean's predictive capacity, but it did prevent us from making statistical comparisons of predictive capacity amongst competing markers of Th2 inflammation via logistic regression because of the risk of over-fitting the model. Although 8 weeks of ICS treatment may be considered a relatively short timeframe, a number of detailed studies of the effects of ICS on FEV_1_ have shown that there is minimal additional response beyond 3-6 weeks [6,7]. Furthermore, reductions in exacerbations and symptoms are likely more relevant to patients and their health-care providers than improvements in FEV_1_. Nevertheless, it was previously shown that a short-term, 6 week improvement in FEV_1_ to ICS was significantly associated with reductions in exacerbations and symptoms over a longer term of 4 months [7]. Ongoing and future longitudinal studies will better establish the performance of the three-gene-mean in predicting longer-term asthma control and exacerbation frequency. Our study also is limited to young adults with mild-to-moderate asthma. The performance of the three-gene-mean in a wider age range, and in patients with severe asthma on high doses of ICS requires further study.

## Conclusions

In summary, we have described a qPCR-based assay of Th2 airway inflammation using RNA derived from epithelial brushings, which we term the three-gene-mean. This three-gene-mean contributes to our understanding of disease mechanisms by identifying Th2 inflammation as significantly associated with AHR in human asthma. By measuring this three-gene-mean and FeNO concurrently we show that they are highly correlated, linking our bronchoscopically obtained measure to a non-invasively obtained biomarker. Finally, we show that the three-gene-mean predicts ICS-response, both in terms of lung function and in patient-oriented measures such as symptoms, yielding ROC curve performance that equals or exceeds that of a set of non-invasive measures. Based on these features, we propose the three-gene-mean as a quantitative standard for measurement of Th2 inflammation in human asthma when epithelial brushings can be obtained. This tool can be applied in clinical research studies for the study of disease mechanisms and assessment of non-invasive biomarkers.

## Abbreviations

AHR: Airway hyper-responsiveness; AUC: Area under the curve; FeNO: Fraction of exhaled nitric oxide; ICS: Inhaled corticosteroids; ROC: Receiver operating characteristic; TGM: Three-gene-mean.

## Competing interests

The authors declare that they have no competing interest.

## Authors’ contributions

NRB conceived and designed the qPCR assay, participated in the collection of samples and subject data in the primary clinical study, analyzed and interpreted the data, and drafted the manuscript. ODS conceived and designed the qPCR assay and analyzed data. CPN coordinated the primary clinical study and participated in the collection of samples and subject data in the primary clinical study. CNN carried out the qPCR and participated in sample processing from the clinical study. JRA conceived and designed the primary clinical study, and revised the manuscript critically for intellectual content. JVF conceived and designed the clinical studies, participated in the collection of samples and subject data in the clinical studies, and revised the manuscript critically for intellectual content. PGW conceived and designed the clinical studies, participated in the collection of samples and subject data in the clinical studies, analyzed and interpreted data, and drafted the manuscript. All authors read and approved the final manuscript.

## Supplementary Material

Additional file 1: Table S1 Primers and probes used in qPCR. **Table S2.** Correlation between epithelial three-gene-mean, POSTN, SERPINB2, CLCA1, and non-invasive markers of inflammation and airway hyperresponsiveness. **Table S3.** Comparative ROC analysis between baseline three-gene-mean and other potential baseline predictors of ICS response. **Figure S1.** Schema of cohorts contributing data to this manuscript. **Figure S2.** Correlation between the centered and scaled qPCR expression values for POSTN, CLCA1, SERPINB2. **Figure S3.** Individual FEV1 responses across 8 week treatment with ICS in subjects with asthma. **Figure S4.** Predictive performance of three-gene-mean and other markers of Th2 inflammation for ICS response at 4 weeks. Click here for file
