# Supplementary material for: A qPCR-based metric of Th2 airway inflammation in asthma
Source: Clin Transl Allergy. 2013 Jul 17;3:24. doi: 10.1186/2045-7022-3-24 (PMC3724712; doi:10.1186/2045-7022-3-24)
Supplement: Additional file 1: Table S1 — Primers and probes used in qPCR. Table S2. Correlation between epithelial three-gene-mean, POSTN, SERPINB2, CLCA1, and non-invasive markers of inflammation and airway hyperresponsiveness. Table S3. Comparative ROC analysis between baseline three-gene-mean and other potential baseline predictors of ICS response. Figure S1. Schema of cohorts contributing data to this manuscript. Figure S2. Correlation between the centered and scaled qPCR expression values for POSTN, CLCA1, SERPINB2. Figure S3. Individual FEV1 responses across 8 week treatment with ICS in subjects with asthma. Figure S4. Predictive performance of three-gene-mean and other markers of Th2 inflammation for ICS response at 4 weeks. [file 2045-7022-3-24-S1.pdf]

Additional File 1

Table S1. Primers and probes used in qPCR

Gene	Type	Sequence
POSTN	outer-forward	GCAAACCACCTTCACGGATCT
	outer-reverse	TTATTCACAGGTGCCAGCAAAG
	inner-forward	CGGATCTTGTGGCCCAATT
	inner-reverse	AGGTGCCAGCAAAGTGTATTCTC
	probe	CTTGGCATCTGCTCTGAGGCC
CLCA1	outer-forward	CCAGGCATTGCTAAGGTTGG
	outer-reverse	ACTGGCCCTGAGAATTGGG
	inner-forward	CCTTGACCCTGACTGTCACGT
	inner-reverse	TTGTTCGTTTTGGAAGTCACTGTAA
	probe	TGCGTCCAATGCTACCCTGCCTC
SERPINB2	outer-forward	CTGAAGTGTTCCACCAAGCCA
	outer-reverse	CAAACTGTGGGCCTCCATGT
	inner-forward	GTGAATGAGGAGGGCACTGAA
	inner-reverse	CCATGTCCAGTTCTCCCTGTC
	probe	TAACACCTCCTGTGCCAGCGGCTG
EEF1A1	outer-forward	TGCTAACATGCCTTGGTTCAAG
	outer-reverse	TTGGACGAGTTGGTGGTAGGAT
	inner-forward	CCTTGGTTCAAGGGATGGAA
	inner-reverse	GCCTCAAGCAGCGTGGTT
	probe	CACTGGCATTGCCATCCTTACGGG
PPIA	outer-forward	ATGAGAACTTCATCCTAAAGCATACG
	outer-reverse	TTGGCAGTGCAGATGAAAAACT
	inner-forward	ACGGGTCCTGGCATCTTGT
	inner-reverse	GCAGATGAAAAACTGGGAACCA
	probe	ATGGCAAATGCTGGACCCAACACA
RPL13A	outer-forward	GGACCGTGCGAGGTATGCT
	outer-reverse	TTCAGACGCACGACCTTGAG
	inner-forward	TATGCTGCCCCACAAAACC
	inner-reverse	TGCCGTCAAACACCTTGAGA
	probe	CAGAGCGGCCTGGCCTCGCT

DNAJA1	outer-forward	AGAGTGCTGTCCCAATTGCC
	outer-reverse	CTTTAGGACTGATCCGCTCCC
	inner-forward	GACCTGGAATGGTTCAGCAAA
	inner-reverse	GACTGATCCGCTCCCCATG
	probe	CCCTGGCACTCCATGCACACAGACT
ACTB	outer-forward	TTGCCGACAGGATGCAGAA
	outer-reverse	GCCGATCCACACGGAGTACTT
	inner-forward	CCTGGCACCCAGCACAAT
	inner-reverse	GCCGATCCACACGGAGTACT
	probe	TCAAGATCATTGCTCCTCCTGAGCGC

Probes used a 5' FAM fluorescent probe with a 3' BHQ quencher.

Table S2. Correlation between epithelial three-gene-mean, POSTN, SERPINB2, CLCA1, and non-invasive markers of inflammation and airway hyperresponsiveness.

	**FeNO** n=19 pre-ICS n=18 post-ICS	**Blood eosinophils** n=24 pre-ICS n=21 post-ICS	**Total serum IgE** n=24 pre-ICS n=20 post-ICS	**PC_{20}** n=24 pre-ICS	**Mean of blood eos.,FeNO,PC_{20}*** n=19 pre-ICS
**three-gene-mean**					
Pre-ICS	r=0.75; p=0.0002	r=0.58; p=0.003	r=0.33; p=0.11	r=-0.65;p=0.0006	r=0.79; p=5.2e-5
Post-ICS	r=0.53; p=0.02	r=0.4; p=0.07	r=0.42; p=0.07	–	–
**POSTN**					
Pre-ICS	r=0.71; p=0.0007	r=054; p=0.006	r=0.43; p=0.037	r=-0.57; p=0.004	r=0.72;p=0.0004
Post-ICS	r=0.56; p=0.02	r=0.26; p=0.25	r=0.30; p=0.20	–	–
**SERPINB2**					
Pre-ICS	r=0.67; p=0.002	r=0.48; p=0.017	r=0.17; p=0.42	r=-0.63; p=0.001	r=0.72;p=0.0004
Post-ICS	r=0.27; p=0.28	r=0.29; p=0.21	r=0.38; p=0.10	–	–
**CLCA1**					
Pre-ICS	r=0.76; p=0.0002	r=0.65; p=0.0006	r=0.33; p=0.11	r=-0.67;p=0.0004	r=0.82; p=1.5e-5
Post-ICS	r=0.56; p=0.02	r=0.51; p=0.02	r=0.40; p=0.08	–	–

FeNO (ppb), blood eosinophils (K/µl), total serum IgE (IU/ml), and PC_{20} (mg/ml) were log-transformed. Gene expression values were centered (by mean) and scaled (by standard deviation) across all samples for that gene.

* Blood eosinophils, FeNO, and $1/PC_{20}$ were log-transformed, and then centered and scaled prior to taking a mean.

Table S3. Comparative ROC analysis between baseline three-gene-mean and other potential baseline predictors of ICS response

	**AUC (number of subjects)**			
	Primary Cohort		Secondary Cohort	
	4 weeks	8 weeks	4 weeks	8 weeks
three-gene-mean	0.94 (23)	0.87 (22)	0.77 (16)	0.91 (15)
POSTN	0.88 (23)	0.81 (22)	0.79 (16)	0.89 (15)
SERPINB2	0.94 (23)	0.88 (22)	0.71 (16)	0.82 (15)
CLCA1	0.88 (23)	0.81 (22)	0.75 (16)	0.86 (15)
blood eosinophils	0.91 (23)	0.84 (22)	0.5 (16)	0.76 (15)
FeNO	0.81 (17)	0.70 (16)	–	–
PC_{20}	0.88 (23)	0.79 (22)	0.43 (16)	0.49 (15)
mean of blood eos., FeNO, PC_{20} *	0.93 (17)	0.82 (16)	–	–
total serum IgE	0.63 (23)	0.58 (22)	0.56 (16)	0.75 (15)

* Blood eosinophils, FeNO, and $1/PC_{20}$ were log-transformed, and then centered and scaled prior to taking a mean.

Figure S1. Schema of cohorts contributing data to this manuscript

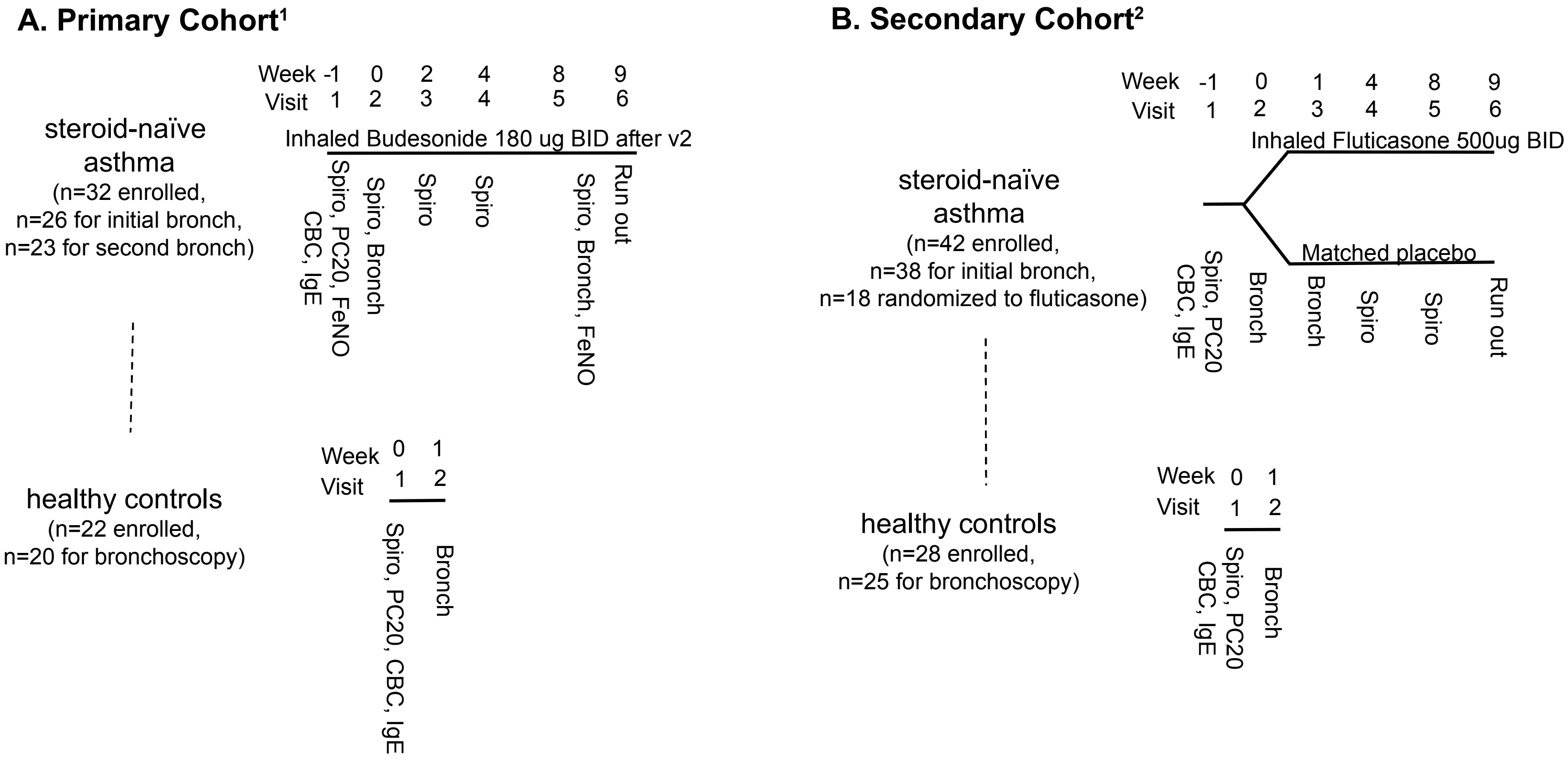

Other findings from these cohorts previously published in:

[1]Solberg OD et al. Airway Epithelial miRNA Expression Is Altered in Asthma. AJRCCM. 2012.

[2]Woodruff PG et al. Genome-wide profiling identifies epithelial cell genes associated with asthma and with treatment response to corticosteroids. PNAS. 2007.

[2]Woodruff PG et al. T-helper Type 2-driven Inflammation Defines Major Subphenotypes of Asthma. AJRCCM. 2009

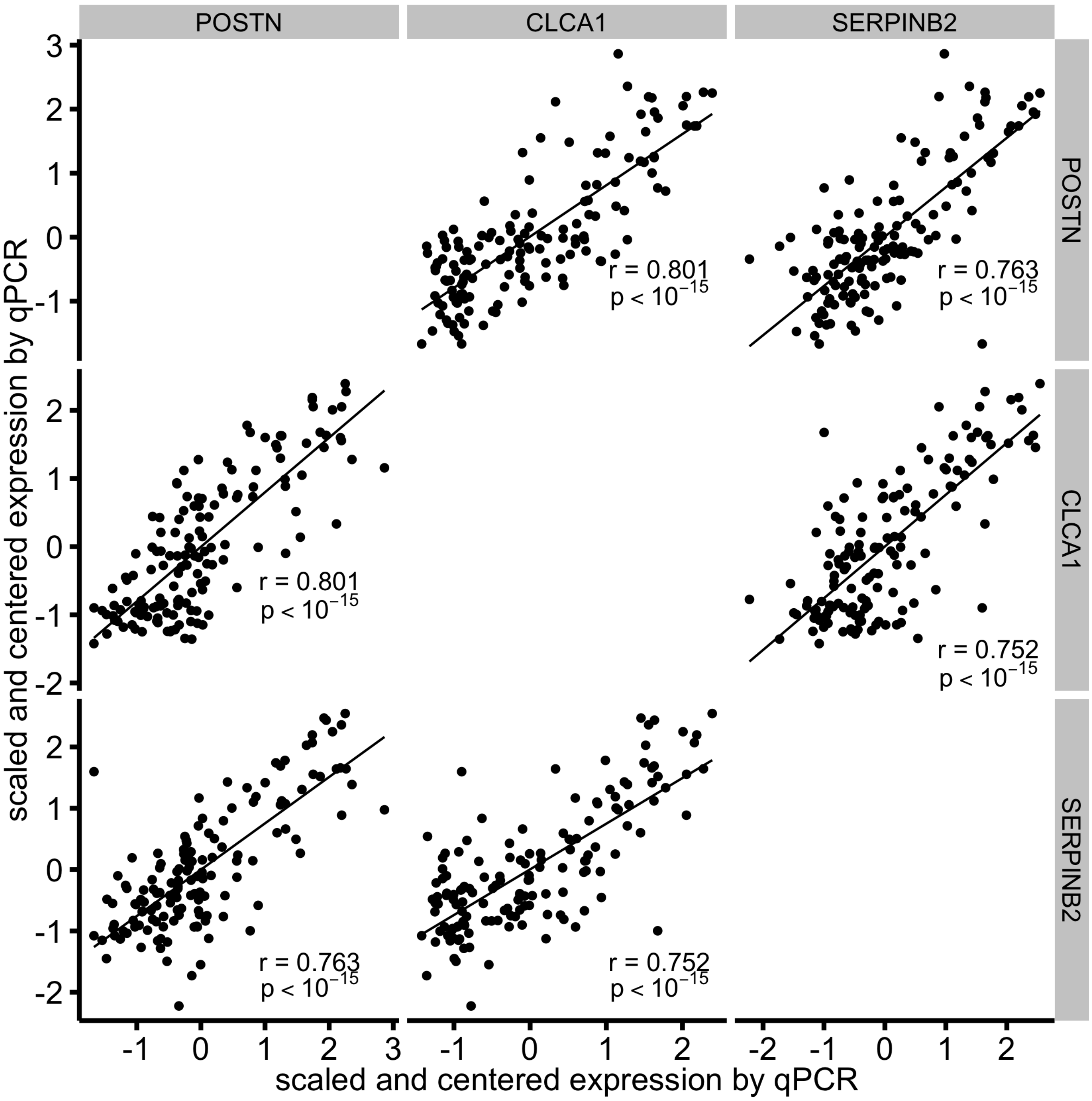

Figure S2. Correlation between the centered and scaled qPCR expression values for POSTN, CLCA1, SERPINB2. Data points are from both the primary and secondary cohorts and include healthy controls, subjects with asthma not on ICS, and subjects with asthma on ICS.

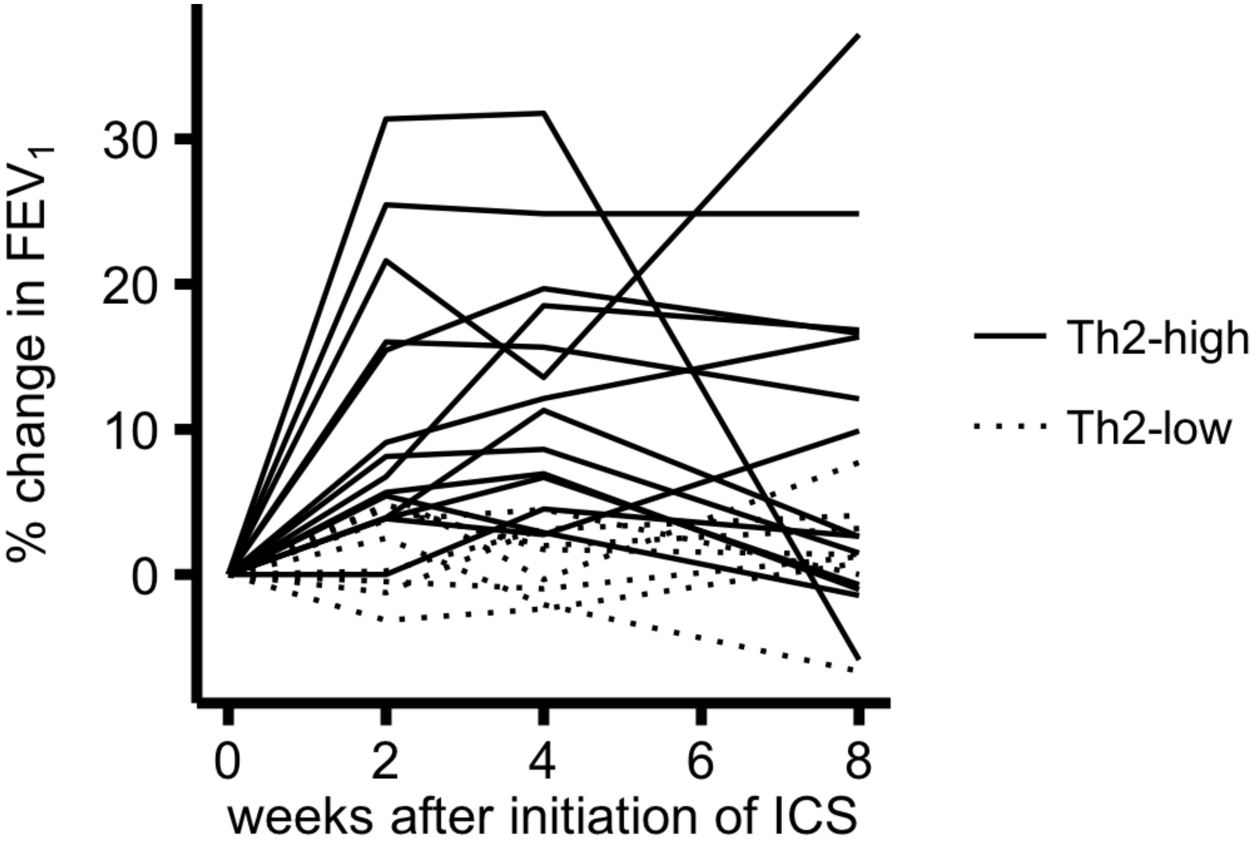

Figure S3. Individual FEV_1 responses across 8 week treatment with ICS in subjects with asthma. Percent change in pre-bronchodilator FEV_1 is relative to week 0. Dotted and solid lines represent Th2-low and Th2-high subjects respectively, with Th2-high defined as a three-gene-mean greater than a value of 0.1.

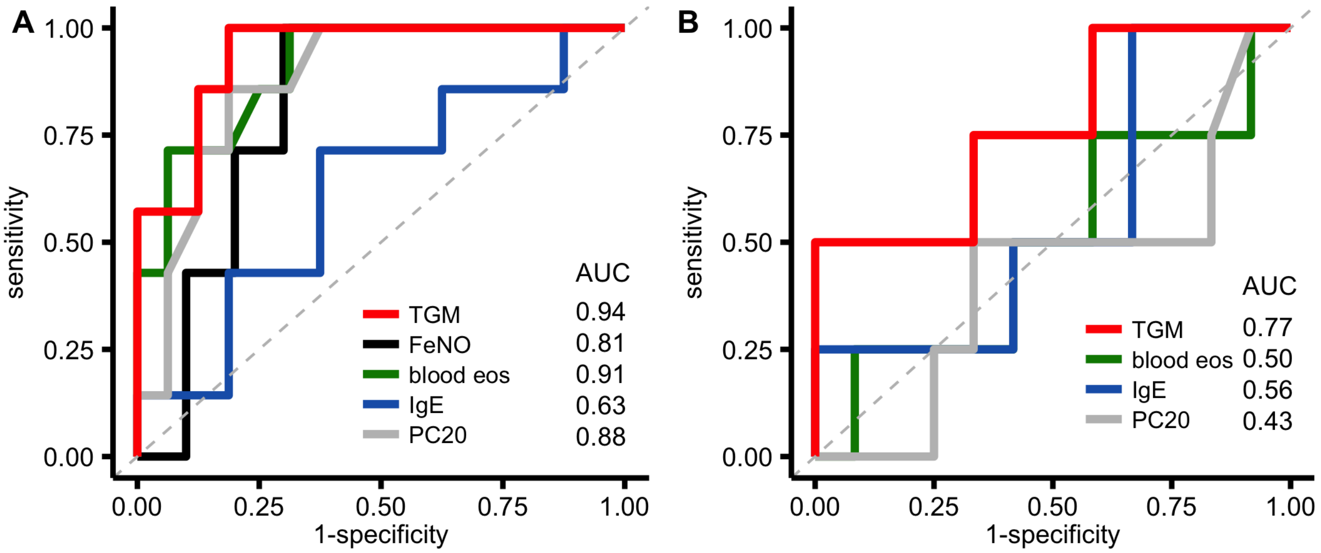

Figure S4. Predictive performance of three-gene-mean and other markers of Th2 inflammation for ICS response at 4 weeks. Receiver operating characteristic (ROC) curves were made to assess the ability of the three-gene-mean and other markers to predict an improvement in FEV_1 after 4 weeks of ICS. Improvement in FEV_1 was defined as an increase of at least 200 ml and 12% relative to week 0. A. ROC curves for baseline measurements of three-gene-mean, FeNO, blood eosinophils, total serum IgE, and PC_{20} to methacholine in the primary study. Seven out of 23 subjects had an improvement in FEV_1. For the three-gene-mean, the cutoff of 0.5 (suggested by the two peaks in Figure 1) has a sensitivity of 100% and a specificity of 69%, a cutoff of 0.1 (mean + 2SD of the healthy distribution) has a sensitivity of 100% and a specificity of 56%, and an alternate cutoff that maximizes the sum of sensitivity and specificity at a three-gene-mean of 0.57 has a sensitivity of 100% and a specificity of 81%. B. ROC curves for the secondary cohort to validate the findings in panel A. Four out of 16 subjects had an improvement in FEV_1. For the three-gene-mean, the cutoff of 0.5 has a sensitivity of 100% and a specificity of 42%, a cutoff of 0.1 (mean + 2SD of the healthy distribution) has a sensitivity of 100% and a specificity of 33%, and an alternate cutoff that maximizes the sum of sensitivity and specificity at a three-gene-mean of 0.57 has a sensitivity of 75% and a specificity of 42%. In both panels, the line of unity is represented by a dashed gray line, corresponding to an AUC of 0.5.
